# Liver necrosis following cholecystectomy in sickle cell disease

**DOI:** 10.1002/ccr3.2820

**Published:** 2020-03-31

**Authors:** Edoardo Maria Muttillo, Emanuele Felli, Patrick Pessaux

**Affiliations:** ^1^ Department of General, Digestive, and Endocrine Surgery University Hospital of Strasbourg Strasbourg France; ^2^ Institut Hospitalo‐Universitaire (IHU) Institute of Image‐Guided Surgery Strasbourg France; ^3^ Department of Surgical Sciences Sapienza University of Rome Rome Italy; ^4^ Institut de Recherche sur les Maladies Virales et Hépatiques Inserm U1110 Université de Strasbourg Strasbourg France

**Keywords:** cholecystectomy, liver failure, liver necrosis, liver surgery, sickle cell disease

## Abstract

Postoperative vaso‐occlusive disease may be a life‐threatening condition in patients affected by sickle cell disease, necessitating sometimes liver transplantation. After laparoscopic cholecystectomy, liver necrosis is usually secondary to intraoperative vascular injury. In this patient, the vaso‐occlusive crisis mimicked a vascular injury with segmental postoperative necrosis.

## Graphical abstract

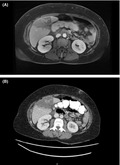

## 1 INTRODUCTION

**Figure 1:**
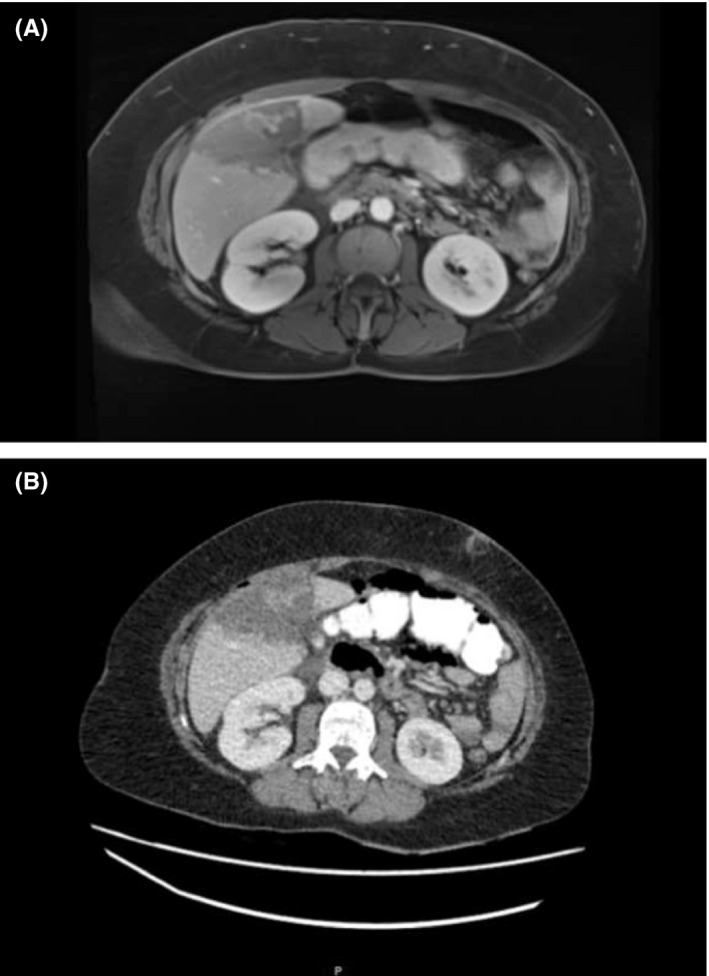
A, MRI venous phase—–a well‐defined necrotic area between segments 4 and 5 as shown in CT scan; margins are more clear delineating an hypointense zone where normal hepatic vein branches are seen. B, CT venous phase—a well‐defined necrotic area between segments 4 and 5 visible as an hypodense zone with clear margins

We present the case of a 30‐year‐old woman who underwent laparoscopic cholecystectomy for gallbladder gallstones. In her past medical history, we notice sickle cell disease (S/S type) for which a bone marrow transplant has been performed in 1991. The patient was admitted for abdominal pain and suspicion of biliary colic in the context of an already‐known gallstone. The biological examinations showed no inflammatory syndrome, normal kidney and hepatic function, and anemia (Hb 8.8 g/dL). No sign of acute cholecystitis was present at imaging; then, laparoscopic cholecystectomy was performed on 31 October 2018. In the second postoperative day, abdominal pain recurred, associated with an important hepatic cytolysis (AST 2851 U/L, ALT 2085 U/L), hyperleucocytosis (WBC 30.000/mL), and fever. An abdominal contrast‐enhanced CT and a liver MRI were performed, and an ischemic area in segments 4 and 5 was noticed (Figure 1A,B); no sign of arterial, portal, or biliary abnormalities was present. Antibiotics were introduced, together with hydration and analgesic therapy. Adequate postoperative hydration, oxygenation, and analgesia are fundamental to prevent this possible life‐threatening condition.^1^, ^2^ The patient recovered progressively with normalization of liver tests and was then discharged in good general condition, with no signs of hepatocellular necrosis.

## CONFLICT OF INTEREST

None declared.

## AUTHOR CONTRIBUTIONS

EMM: Author of the image article. Treatment of the patient. EF: Author of the image article. Treatment of the patient. PP: Department head, data presentations. Treatment of the patient.
